# Egyptian Rousette IFN-ω Subtypes Elicit Distinct Antiviral Effects and Transcriptional Responses in Conspecific Cells

**DOI:** 10.3389/fimmu.2020.00435

**Published:** 2020-03-13

**Authors:** Stephanie S. Pavlovich, Tamarand Darling, Adam J. Hume, Robert A. Davey, Feng Feng, Elke Mühlberger, Thomas B. Kepler

**Affiliations:** ^1^Department of Microbiology, Boston University School of Medicine, Boston, MA, United States; ^2^National Emerging Infectious Diseases Laboratory, Boston University, Boston, MA, United States; ^3^Department of Virology and Immunology, Texas Biomedical Research Institute, San Antonio, TX, United States; ^4^Department of Mathematics and Statistics, Boston University, Boston, MA, United States

**Keywords:** interferon omega, bat, Egyptian rousette, interferon stimulated genes, antiviral response, Marburg virus

## Abstract

Bats host a number of viruses that cause severe disease in humans without experiencing overt symptoms of disease themselves. While the mechanisms underlying this ability to avoid sickness are not known, deep sequencing studies of bat genomes have uncovered genetic adaptations that may have functional importance in the antiviral response of these animals. Egyptian rousette bats (*Rousettus aegyptiacus*) are the natural reservoir hosts of Marburg virus (MARV). In contrast to humans, these bats do not become sick when infected with MARV. A striking difference to the human genome is that Egyptian rousettes have an expanded repertoire of IFNW genes. To probe the biological implications of this expansion, we synthesized IFN-ω4 and IFN-ω9 proteins and tested their antiviral activity in Egyptian rousette cells. Both IFN-ω4 and IFN-ω9 showed antiviral activity against RNA viruses, including MARV, with IFN-ω9 being more efficient than IFN-ω4. Using RNA-Seq, we examined the transcriptional response induced by each protein. Although the sets of genes induced by the two IFNs were largely overlapping, IFN-ω9 induced a more rapid and intense response than did IFN-ω4. About 13% of genes induced by IFN-ω treatment are not found in the Interferome or other ISG databases, indicating that they may be uniquely IFN-responsive in this bat.

## Introduction

Bats comprise about 20% of all classified mammal species with over 1,200 species and host a number of viruses known to cause severe disease in humans. While humans develop severe and life-threatening illnesses from many of these viruses (e.g., henipaviruses, SARS and MERS coronaviruses, and filoviruses), bats show no symptoms of disease in natural or experimental infections (1, 2). The adaptations (in host or virus) that allow bats to host emerging viruses without developing symptoms of disease are not yet known.

Type I interferons (IFNs) are an important component of the early antiviral immune response, and make up a diversified multi-gene family, including subtypes like α, β, δ, ω, ε, and others (3). Type I IFNs are induced by the recognition of viral pathogen-associated molecular patterns (PAMPs), and act by inducing interferon stimulated genes (ISGs) that collectively contribute to an antiviral response (4, 5). All type I IFNs bind to and signal through the same heterodimeric receptor complex IFNAR1/2, but both evolutionary analyses and functional studies suggest that multiple IFN subtypes make non-redundant contributions to immunity (6–10). Although the exact functional contribution for each IFN is not completely understood, differences in the interaction of various IFN subtypes with IFNAR1/2 are known to differentially induce downstream ISGs (10–12). As a result, differences in pathogen-specific antiviral effect are possible, depending on the amount and profile of ISGs induced by a particular IFN.

The importance of type I IFNs in innate antiviral responses and in bridging innate and adaptive immune responses has sparked interest in exploring this pathway in several bat species. Due to the lack of bat-derived IFNs, much of the work to analyze IFN responses in bats thus far has been done using universal interferon (UIFN; a pan-species type I IFN derived from two human IFN-α subtypes) or cell culture medium from stimulated bat cells as a surrogate for authentic bat IFN (13–15). More recently, bat IFN responses have been explored using recombinant bat IFN-α or –β, but additional bat IFN subtypes remain poorly characterized (16–18).

We have previously shown that the type I IFN locus is expanded in the Egyptian rousette (*R. aegyptiacus*), an asymptomatic host of Marburg virus (MARV) (19). Whereas, humans have a single IFNW gene, almost half of the Egyptian rousette IFN genes belong to the IFN-ω subtype. The functional relevance of the expansion is not known. In humans and other species, IFN-ω is induced by viral infection and has potent antiviral activity against various RNA viruses, including vesicular stomatitis virus (VSV), bovine viral diarrhea virus, yellow fever virus, West Nile virus, and influenza A virus (20–24). Multiple subtypes of porcine IFN-ω are expressed after viral infection and have dramatic differences in activity despite very few single nucleotide polymorphisms (23). The ISGs induced specifically by these IFN-ω proteins, however, are not known. To begin to understand the role of these genes in the immune response to viruses in bats, we synthesized and purified recombinant Egyptian rousette IFN-ω proteins. We characterized the antiviral potency and efficacy of these recombinant proteins against VSV and against MARV, and examined the downstream ISGs they induce.

## Results

### Phylogenetic Structure of the IFN-ω Subfamily in the Egyptian Rousette

**Figure 1:**
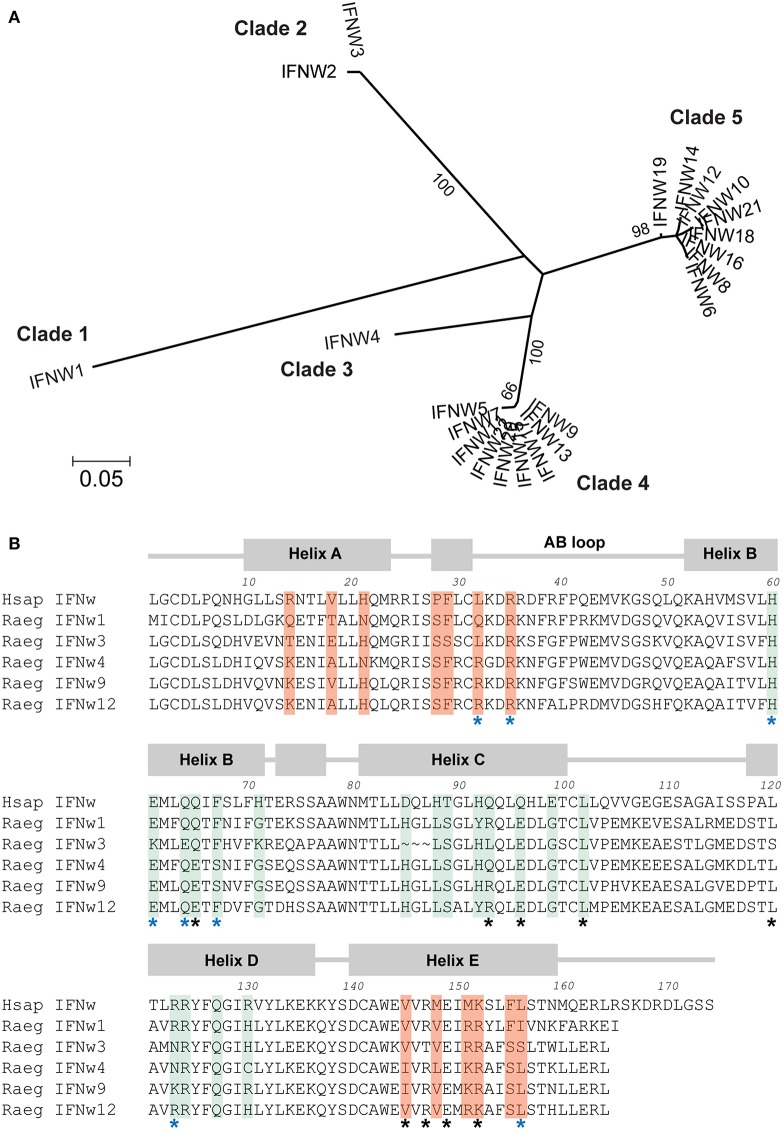
Comparison of five clades of Egyptian rousette IFN-ω proteins. **(A)** Phylogenetic tree of Egyptian rousette IFN-ω proteins. A maximum likelihood tree of Egyptian rousette IFN-ω proteins was constructed in RAxML and formatted in MEGA v7. Bootstrap evidence (percentage of 100 bootstrap replicates) is labeled on branches if over 65. **(B)** Multiple sequence alignment of representative bat IFN-ω proteins showing conserved and divergent putative receptor binding residues. Predicted signal sequences were cleaved for each protein. Annotations and putative receptor binding sites are based on the structure of the human IFN-ω-IFNAR1/2 complex (12). Residues important for interacting with IFNAR1 are highlighted in green, and residues that interact with IFNAR2 are highlighted in orange. Black stars indicate conserved residues that help anchor human IFNs to receptor subunits, and blue stars indicate conserved residues that influence the energetics of receptor binding. All residues highlighted as interacting with IFNAR1 or IFNAR2 but without stars are considered “ligand-specific” according to the model in Thomas et al. (12).

Although the Egyptian rousette IFN-ω subfamily has 22 members, many of these genes fall into clusters of highly similar genes. Phylogenetic analysis shows that the 22 IFNs are divided into five distinct groups: two large clades, each containing IFNs with >0.847 amino acid pairwise identity, one pair of IFNs sharing 0.989 identity with each other and <0.681 with any other, and two single IFNs with maximum homology of 0.832 to any other (Figure 1A). The IFNAR1 and IFNAR2 binding sites in all human type I IFNs, including IFN-ω, have been well-characterized (12). We annotated the broad collection of residues that participate in receptor binding on all 22 proteins using the NCBI conserved domain database search. While proteins within a clade have high identity at these receptor-binding sites, these sites are much less conserved between clades (Table 1).

**Table 1:** Percent identity (amino acid) at all potential receptor binding sites between and within Egyptian rousette IFN-ω clades.

**Clade**	**1**	**2**	**3**	**4**	**5**
1	–	–	–	–	–
2	59.6	95.7	–	–	–
3	68.1	63.8	–	–	–
4	70.2	66.0	80.9–83.0	95.7–100	–
5	72.3–76.6	59.6–61.7	74.5–78.7	78.7	95.7–100

*Each entry shows the percent amino acid identity between members of different clades at IFNAR1 and IFNAR2 binding sites. Percent identity is calculated as the total number of differences divided by the total number of binding sites. If multiple percent identities are shown, the values represent the minimum and maximum percent identities between individual clade members*.

Although all human type I IFNs bind to the same receptor complex, their downstream signaling upon binding differs among types and subtypes. Crystal structure analysis of two human type I IFNs bound to their receptor has led to a model that connects differences in receptor recognition and conformation to differences in downstream signaling (12). According to this model, ligand residues that interact with the receptor are classified into three groups. First, there are conserved “anchor” residues—those that are identical among all or most human IFN subtypes. Second, there are conserved “modulating” residues that are identical among all or most human IFN subtypes but when mutated, change the energetics of the ligand-receptor interaction and lead to functional changes. Third, there are “ligand-specific” residues that vary greatly among human IFN subtypes (12). We examined several rousette IFN-ω subtypes using this classification and compared residues that were identified as functionally important. The “anchor,” “modulating,” and “ligand-specific” residues are overall fairly conserved between the human and at least one of the bat IFN-ω proteins (Figure 1B). However, when comparing the bat proteins, there is noticeable diversity among the “ligand-specific” residues. Additionally, by definition, conservation of the “modulating” residues does not guarantee an energetically equal reaction with the receptor subunits. Together, this suggests that the five rousette IFN-ω groups are likely to react with the receptor with different kinetics and affinities, potentially resulting in different downstream effects.

### Egyptian Rousette IFN-ω Proteins Are Functional and Exhibit Different Antiviral Potencies

To examine whether Egyptian rousette IFN-ω proteins retain the canonical function of type I IFNs as antiviral proteins, we expressed two recombinant IFN-ω proteins (rIFN-ω4, rIFN-ω9) containing a C-terminal histidine tag (6x-His) in 293F cells and purified the proteins from cell supernatants as previously described (19). As a negative control, we included an unrelated 6x-His-tagged protein (rD1) of similar size. We tested the antiviral efficacy of these recombinant proteins against VSV, using UIFN as a positive control.

**Figure 2:**
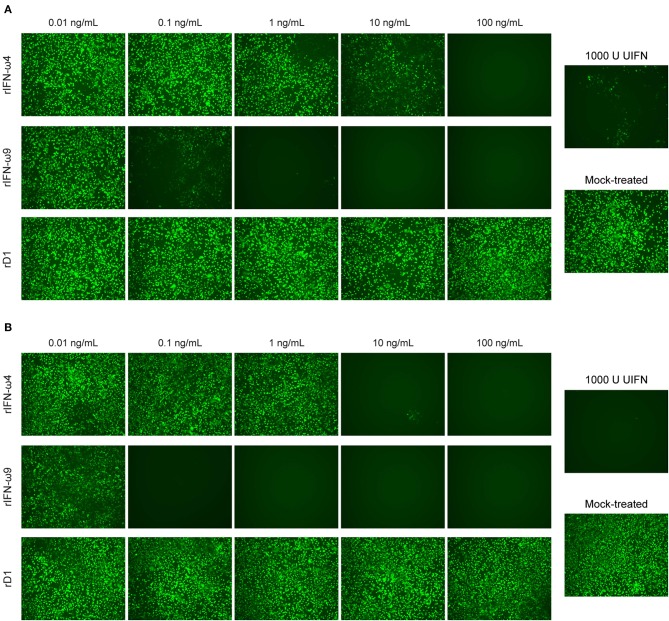
Antiviral effect of recombinant Egyptian rousette IFN-ω4 and IFN-ω9. RoNi/7.1 cells seeded in 96-well plates were mock-treated or treated with different concentrations of the purified proteins (0.01–100 ng/mL) for **(A)** 4 or **(B)** 8 h and then infected with VSV containing an additional transcriptional unit encoding eGFP (VSV-eGFP) at a multiplicity of infection (MOI) of 0.1. As a positive control, cells were treated with 1,000U of UIFN prior to infection. Cells were imaged for eGFP expression 1 day post infection on a fluorescent microscope at 10x magnification. Images are representative of at least two independent experiments.

As expected, VSV replication was not inhibited in untreated cell or cells treated with rD1, whereas there was significant inhibition of VSV replication in cells pretreated with UIFN (Figure 2). Although both rIFN-ω4 and rIFN-ω9 showed antiviral activity against VSV, this effect was more pronounced for rIFN-ω9, which was effective at concentrations a 100-fold less than IFN-ω4 after 4 h treatment, and even lower concentrations after 8 h treatment (Figure 2).

### rIFN-ω Treatment of RoNi/7.1 Cells Results in a Concentration- and Time-Dependent ISG Expression Profile

**Figure 3:**
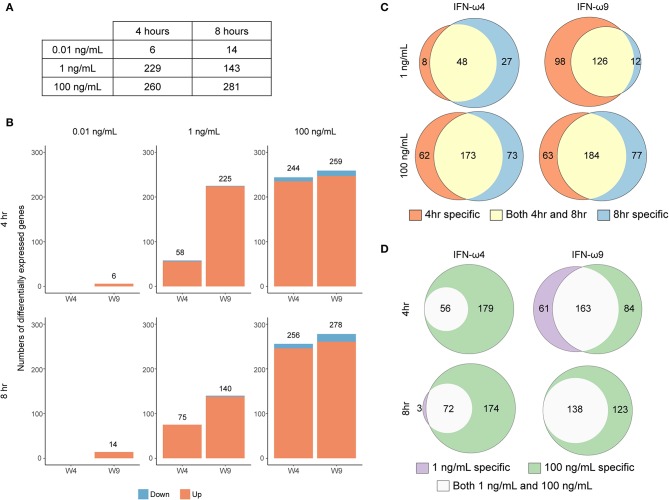
Concentration- and time-dependent differential expression after rIFN-ω treatment. **(A)** The total number of genes that rejected the null in the six ANOVA-like tests. **(B)** The total number of DEG under each treatment compared to control after pairwise comparisons of genes that passed significance criteria in the ANOVA-like test and *p* ≤ 0.05/3 in the pairwise test. Impact of time and concentration on number of differentially expressed genes for a given rIFN-ω. Venn diagrams showing the overlap in differentially expressed genes between **(C)** different treatment times or **(D)** different concentrations of a given rIFN-ω. Only upregulated genes were included.

We treated RoNi/7.1 cells with UIFN, or three different concentrations of rIFN-ω4, rIFN-ω9, or rD1 for 4 or 8 h and collected RNA for mRNA sequencing (RNA-Seq) and differential gene expression analysis. We first compared the effect of treatment on mean expression of each gene via an ANOVA-like test for differential expression. For each gene rejecting the null in the ANOVA analysis (ANOVA FDR < 0.05) (Figure 3A), every IFN treatment condition was contrasted with the appropriate control treatment; rIFN-ω-treated samples were compared to rD1-treated samples at the same concentration and time point. UIFN-treated samples were compared to untreated samples at the same time point in a pairwise analysis. The total numbers of genes that passed our pairwise reporting criteria (2-fold expression change or greater and Bonferroni-corrected *p* < 0.05) are shown in Figure 3B and Table S1.

Most of the differentially expressed genes were upregulated relative to the corresponding control, and there were very few downregulated genes across all conditions. This trend of positive gene expression has also been seen with UIFN treatment of cells from the black flying fox (*Pteropus alecto*) (14). IFN concentrations that were not observed to be antiviral in our VSV-eGFP assay induced very few genes (Figures 2, 3B). For example, 0.01 ng/mL of rIFN-ω9 induced only six genes after 4 h and 14 genes after 8 h (Table S1). In contrast, IFN concentrations that blocked VSV-eGFP replication induced many more genes. For a given concentration, many genes were induced at both time points (Figure 3C). At a given time point, almost every gene induced at a low IFN concentration was also induced at a high concentration, with the main exception being 4 h rIFN-ω9 treatment, where 61 genes were induced by 1 ng/mL but not by 100 ng/mL (Figure 3D and Table S1).

### rIFN-ω9 Induces the Expression of Many More ISGs Than Does rIFN-ω4

**Figure 4:**
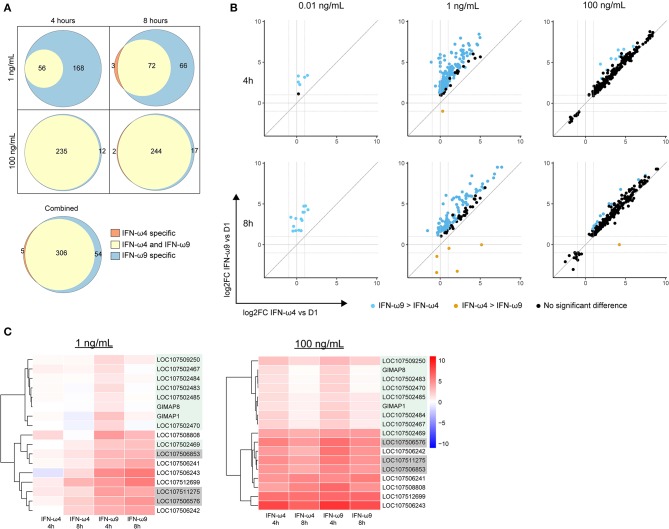
Comparing genes induced by rIFN-ω4 and rIFN-ω9 across time of treatment and concentration. **(A)** Each Venn diagram shows the overlap in differentially expressed genes (ANOVA FDR ≤ 0.05, pairwise log_2_ fold change > 1, pairwise *p* ≤ 0.05/3) for rIFN-ω4 and rIFN-ω9 treated samples at a given concentration and time. The combined diagram shows the overlap between genes that were differentially expressed at any concentration or time for rIFN-ω4 and rIFN-ω9 treated samples. Only upregulated genes are shown. **(B)** The relative log_2_ fold change (compared to rD1 treatment) of genes that were differentially expressed by ANOVA analysis in samples treated with rIFN-ω4 or rIFN-ω9. Only genes with FDR ≤ 0.05 are shown. The color of each point indicates the result of a pairwise test with the null hypothesis that rIFN-ω4 and rIFN-ω9 expression were the same. Blue points are genes that rejected the null with significantly higher expression after rIFN-ω9 treatment than after rIFN-ω4 treatment. Orange points also rejected the null, but indicate a higher rIFN-ω4-induced expression compared to rIFN-ω9. **(C)** IFN-induced GTPases induced by rIFN-ω treatment over time. Putative GIMAPs are highlighted in green, putative GVINs are highlighted in gray, and the remaining genes are putative GBPs.

Given the observed difference in antiviral activity between IFN-ω4 and IFN-ω9, we next compared the ISG expression profile induced by each IFN (Figure 4 and Table S1). At both time points, 1 ng/mL of IFN-ω9 induced many more genes than 1 ng/mL of IFN-ω4, and there were very few genes induced only by IFN-ω4 (Figure 4A). A higher concentration of IFN-ω4 induced additional genes, though only two of these genes were unique to IFN-ω4 treatment. When all time points and concentrations were combined, IFN-ω4 treatment induced only five unique genes, while IFN-ω9 treatment induced 54 unique genes.

We next compared the expression levels of the genes induced by both IFNs by performing a pairwise comparison between expression in IFN-ω4 and IFN-ω9 treated samples at a given time point and concentration (Figure 4B). At a low concentration of 1 ng/mL, IFN-ω9 treatment resulted in greater expression of all the genes that were induced by both IFN-ωs at both 4 and 8 h of treatment. In contrast, at a high concentration of 100 ng/mL, the change in the expression ratio was similar between IFN-ω4 and IFN-ω9 treatments (Figure 4B). This suggests that at high concentrations, the ISG response between the two IFNs may be interchangeable.

We also examined the change in expression over time at a given concentration of IFN (Figure S1). At a low concentration, genes induced by IFN-ω4 appeared to be increasing in expression over time. In contrast, genes induced by IFN-ω9 began at a higher expression level at 4 h, and many genes had reduced expression at 8 h, suggesting that a peak response may have already been achieved. At a high concentration, the kinetic profiles of both IFN-ωs were very similar, and many genes had lower expression at 8 h than at 4 h. This is consistent with an early peak response, followed by subsequent downregulation, though additional time points and concentrations would be needed to examine the kinetics in detail.

### IFN-ω Proteins Induced Novel and Known ISGs

**Figure 5:**
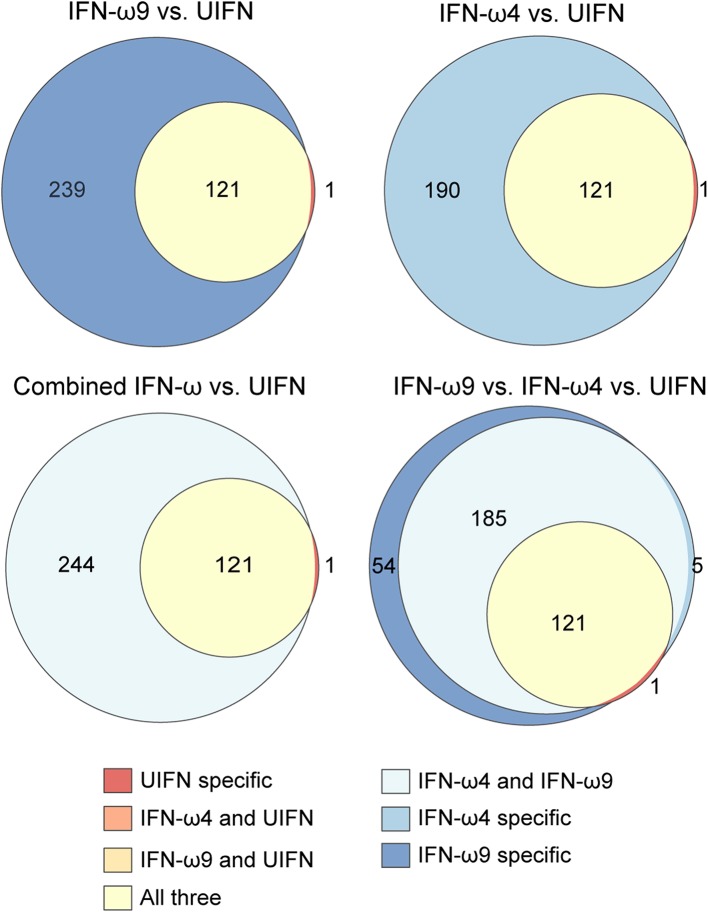
Egyptian rousette IFN-ω proteins induce many more genes than UIFN. Venn diagrams show the overlap in genes induced by rIFN-ω and UIFN. Only upregulated genes were included. Combined IFN-ω refers to genes that were differentially expressed in any rIFN-ω treated sample (any concentration, any time point, either rIFN-ω4 or–rIFN-ω9).

To explore whether additional IFNs may provide a host advantage by inducing unusual ISGs, we compared genes induced by IFN-ωs and UIFN (Figure 5). Both IFN-ωs and UIFN induced a familiar panel of ISGs, including pathogen sensors (DDX58, IFIH1, CGAS, ZBP1), and antiviral ISGs like IFIT1, IFIT2, Mx genes, ISG15, and OAS genes. As part of a positive-feedback loop, IFN treatment upregulates the expression of interferon regulatory factor (IRF) genes that are transcription factors for further IFN induction. Both IFN-ωs induced several IRFs, including IRF1, 2, 4, 7, 8, and 9, while UIFN induced only IRF4, 7, and 9.

We compared the genes induced by each IFN-ω and UIFN with those in multiple ISG databases to determine how many of them are known to be type I IFN-inducible (Table 2 and Table S2). Since these databases are composed of data from other species (27), we excluded any MHC genes from the analysis as these gene evolve in complex ways and homology is inherently uncertain among species. We cross-referenced the remaining genes with data from (1) the Interferome—a database of ISGs from a wide variety of human and mouse studies (27); (2) data from a recent analysis of the IFN response in ten different species (25); and (3) ImmGen—a database of ISGs from a variety of human immune cells (26). In our hands, more than 95% of genes induced by UIFN were found in the at least one of these three databases. In contrast, of the 358 genes upregulated by either IFN-ω at any time point or concentration, 87.4% (313 genes) were found in at least one database, and 12.6% (52 genes) were not. These percentages are slightly lower than those found in similar studies with UIFN and recombinant IFN-α3 in the black flying fox (14, 17). This is partly because we use multiple databases that capture ISGs across a number of species and cell types and partly because there are differences in the total number of upregulated genes in those studies.

**Table 2:** Known and uncharacterized ISGs.

	**Number of genes in any ISG database (percent of total)**	**Number of genes not in any ISG database (percent of total)**	**Total number of upregulated genes^a^**	**Uncharacterized genes**
UIFN	115 (95.8)	6 (4.2)	121	2
IFN-ω4	268 (86.7)	43 (13.3)	309	21
IFN-ω9	312 (88.4)	48 (11.6)	353	20
Both IFN-ωs	313 (87.4)	52 (12.6)	358	22

*Only upregulated genes were searched against the Interferome database, data from Shaw et al. (25), and data from Mostafavi et al. (26). “Both IFN-ωs” refers to genes upregulated after treatment with either IFN-ω at any concentration and time point. Genes that were annotated as uncharacterized by the NCBI annotation pipeline were labeled as uncharacterized genes, and were included in the total count*.

a *MHC class I-like or class II-like genes were excluded since the naming structure of these genes can be species-specific (UIFN: 1 gene, IFN-ω4: 2 genes, IFN-ω9: 7 genes, “Both IFN-ωs”: 7 genes)*.

Of the 52 genes not previously known to be IFN-inducible, 22 were completely uncharacterized by the NCBI annotation pipeline. However, given that the Egyptian rousette genome was itself annotated by this pipeline, which uses all available genomes in GenBank and RefSeq to produce annotations, a more comprehensive examination will be required to determine whether the previously uncharacterized ISGs may be genes thus far unique to the Egyptian rousette.

Many of the 313 genes known to be IFN-inducible were paralogs of canonical ISGs, especially GTPase-related families. Among these genes were Mx genes, guanylate binding proteins (GBPs), GTPase IMAP family members (GIMAPs), and interferon-induced very large GTPases (GVINs). Humans have two functional Mx genes and seven functional genes each in the GIMAP and GBP subfamilies. GVIN1 is an interferon-inducible gene in other species but is only a pseudogene in the human genome. IFN-ω4 or -ω9 treatment of RoNi/7.1 cells led to the induction of nine different GIMAPs, five GBPs, and three GVIN genes (Figure 4C and Table S1). In contrast, UIFN induced only three interferon-inducible GTPases other than Mx genes suggesting that IFN-ωs may be able to induce a more diverse ISG response by tapping into expanded families of GTPase-related genes. Consistent with this hypothesis, the bat IFN-ωs induced all but one of the ISGs that were induced by UIFN, as well as additional ISGs that were not induced by UIFN (Figure 5). Fifty four genes were expressed only with IFN-ω9 treatment, while five were induced only with IFN-ω4 treatment. These results indicate that UIFN does not appropriately represent the ISG response of bat-specific IFNs.

It has been reported that IFN-α treatment of black flying fox cells led to high expression of RNASEL, an RNase that degrades cellular and viral RNA after activation via the 2′,5′-oligoadenylate synthetase (OAS) family of nucleic acid sensors (14, 17, 28). Surprisingly, neither UIFN nor IFN-ωs led to RNASEL induction in our experiments. This may be due to species, cell-type, or treatment time and concentration differences. A higher basal (rather than inducible) expression of RNASEL was observed in Egyptian rousette cells (29), which could further support the hypothesis of a species-specific difference among bats.

There are a number of ISGs that were shown to have antiviral activity against filoviruses, including tetherin, Zinc finger antiviral protein (ZAP), interferon-induced transmembrane (IFITM) proteins, and ISG15 [reviewed in (30)]. While ZAP was not induced under any condition, ISG15 expression was induced by both IFN-ωs and UIFN, and tetherin and IFITM3 were only induced by IFN-ω treatment.

### IFN-ω Proteins Inhibit MARV *in vitro*

**Figure 6:**
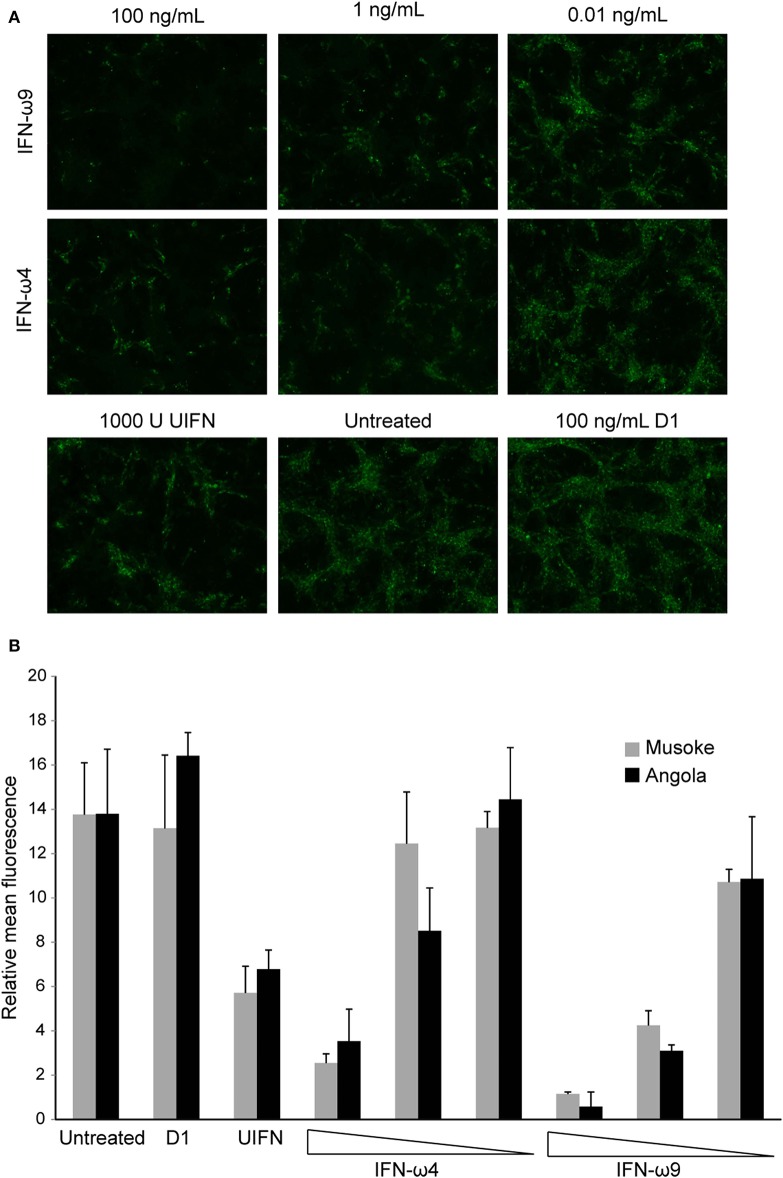
Egyptian rousette IFN-ω proteins protect RoNi cells from MARV infection. RoNi cells seeded in 96-well plates were mock-treated or treated with varying amounts of rIFN-ω4 or rIFN-ω9, 100 ng/mL of rD1, or 1,000U of UIFN for 18 h and infected with MARV Musoke or MARV Angola at an MOI of 3. One day post infection, cells were fixed and immunofluorescence analysis was performed with an anti-MARV nucleocapsid antiserum. Cells were imaged for fluorescent signal at 10x magnification. **(A)** Cells infected with MARV Angola at 1 day post infection. Images are representative of two independent experiments. **(B)** Fluorescent signal of pictures (two per sample per experiment) from two independent experiments was quantified in ImageJ. Error bars represent standard error of the mean.

To determine whether IFN-ω proteins could protect against MARV infection, RoNi/7.1 cells were treated with rIFN-ω4, rIFN-ω9, rD1, or UIFN for 18 h and infected with MARV Angola or MARV Musoke at an MOI of 3. One day post infection, the cells were fixed and the infection rate was quantified by immunofluorescence. Consistent with the results of the VSV bioassay, both IFN-ω4 and IFN-ω9 significantly inhibited MARV replication, with IFN-ω9 exhibiting greater antiviral activity. Surprisingly, treatment with UIFN had much less of an antiviral effect than either IFN-ω (Figure 6).

## Discussion

Although the number of type I IFN genes appears to vary substantially among bat species, many bat genomes encode multiple IFN subtypes beyond IFN-α and –β. These additional genes include single copies of IFN-ε and IFN-κ, which have orthologs in humans, as well as multiple copies of subtypes that exist only in one copy (IFN-ω) or not at all (IFN-δ) in humans (31, 32).

In humans, the production and secretion of IFN-ω is induced by viral infection, and human IFN-ω is associated with more potent anti-proliferative and antiviral capabilities than other type I IFNs (21, 24, 33, 34). Based on previous observations of the large expansion in the type I IFN-ω subfamily in the Egyptian rousette (19), we sought to examine members of this family to gain insight into the contributions of these proteins to Egyptian rousette antiviral immunity and to ask whether the considerable duplications in the IFN locus may have functional relevance for antiviral responses. In species with multiple IFN-ω genes, like *Sus scrofa* (pig) and *Bos taurus* (cow), differences in induction and antiviral potency among IFN-ω paralogs have been observed (23, 35–37). Consistent with this work, we found functional differences between two rousette IFN-ω subtypes, supporting the hypothesis that the IFN-ω subtypes are not interchangeable.

Differences in antiviral potency among tested IFNs were dramatic, with 100-fold differences between IFN-ω4 and IFN-ω9. These differences are not explained by a difference in sample purity, since the technique used for isolating recombinant protein yielded preparations that were remarkably pure (Figure S2). In general, longer exposure to IFN prior to infection resulted in greater antiviral efficacy even at low IFN concentrations. This could be explained by a second wave of IFN induction due to positive feedback or by higher concentrations of antiviral ISGs.

At low concentrations, IFN-ω9 induced more genes and higher expression levels of the same genes when compared to IFN-ω4, which may explain their differences in potency. However, at high concentrations these proteins induced very similar though not identical transcriptional responses. The high overlap in differentially expressed genes and similarity in level of gene expression between IFN-ω4 and -ω9 suggest that these proteins could be redundant at high concentrations. Nevertheless, both proteins induced a number of unique genes, and these were furthermore distinct from those induced by UIFN. These data reinforce the notion of IFN subtype-specific differences, and highlight the importance of using bat-specific IFNs for understanding bat ISG responses.

Among the genes uniquely induced by IFN-ω proteins were multiple paralogs of known ISGs, especially interferon stimulated GTPases, including Mx, GVIN, GBP, and GIMAP genes. Similar to their counterparts in other species, bat Mx genes limited viral replication in *in vitro* studies (38). The GVIN family is reduced to a single pseudogene in humans, but GVIN genes are highly expressed in mice after IFN stimulation, though their antiviral function remains uncharacterized (39, 40). The presence and IFN-dependent upregulation of multiple distinct GVINs in the Egyptian rousette and in other bats suggest that these genes do play an antiviral role in bats (14, 17).

GBPs are induced by type I, type II, and type III IFNs, and are mainly known for their GTPase-dependent role as cell-autonomous defenders against bacterial and protozoal infection (41). However, several members of the GBP family in humans and/or mice have been shown to have antiviral activity against VSV, influenza A virus, encephalomyocarditis virus, and retroviruses, including HIV-1 (40–42). GBPs are recruited to pathogen-containing compartments, including viral replication sites, by autophagy related proteins (43). Once recruited, they exert a variety of antiviral activities that interfere with various steps of the viral replication cycle within these compartments, coordinate lysis or lysosomal fusion, and activate the inflammasome (44, 45). For example, GBP1 inhibits the delivery of Kaposi's sarcoma-associated herpesvirus virions to the nucleus by interfering with actin filament organization (46). This blocking mechanism could be significant for MARV infection, given that MARV also relies on actin filaments to transport nucleocapsids to the budding sites (47). Given their close association with autophagy related proteins, GBPs can direct autophagy for pathogen clearance (45), which has recently been shown to be one mechanism by which bats can limit viral infection (48).

There is also compelling evidence that GBPs act in concert, as hetero- and homodimers, and non-redundantly against different viral and bacterial pathogens (49, 50). If this phenomenon is also present in bats, the induction of 5 different GBPs by IFN-ω subtypes could mean a second tier of antiviral flexibility. In addition to these varied functions, GBPs play a role in inflammasome activation, either by helping to create PAMPs by lysing pathogen-containing compartments or by promoting caspase-11 activation or both (40). Fewer inflammasome-related genes and a diminished NLRP3-related inflammasome response have been observed in bats compared to other mammals (51, 52); whether the expression of multiple GBPs could compensate for this diminished response with less inflammation remains to be explored.

GIMAPs are involved in the development, maintenance, and homeostasis of lymphocytes, especially CD4^+^ T cells and B cells, but also T regulatory cells (Tregs) (53–55). In the absence of individual GIMAP members, there is progressive lymphocyte loss leading to lymphopenia, poor cell proliferation, and paradoxical autoimmune states because of impaired Treg function (54–58). A previous gene family analysis showed that the GIMAP family is expanded in the Egyptian rousette compared to a bat ancestor (19), with 14 putative members. It is striking that nine of the 14 GIMAP genes are induced by IFN-ω treatment, with only a single GIMAP induced by UIFN treatment. GIMAPs are also expressed in black flying fox cells after UIFN or bat IFN-α stimulation (14, 17), although not as many individual GIMAP genes were observed to be upregulated in these studies. Given the classical role of these genes in B and T cells, it will be important to examine their induction in bat lymphocytes once reagents are available for this work.

We show that recombinant IFN-ω proteins qualitatively inhibit MARV infection *in vitro*, with noticeable differences in their antiviral potency. Kuzmin et al. have previously shown that two Egyptian rousette cell lines transfected with either Egyptian rousette IFN-β or a consensus IFN-α are able to resist both EBOV and MARV infection (59), but whether filovirus infection itself induces IFNs in bat cells is unclear. In general, MARV infection seems to suppress immune gene expression in immortalized Egyptian rousette cells, yet there are conflicting studies regarding the extent of this suppression. The EBOV antiviral protein VP35 is known to efficiently inhibit IFN induction *in vitro* in human cells; in contrast, MARV VP35 is a much weaker inhibitor, and MARV infection of human THP-1 cells does lead to an IFN response (60, 61). Infection of RoNi/7.1 cells with a MARV isolate from a wild-caught bat did not lead to any IFN induction at 3, 8, or 24 h after infection, and in fact, led to very little antiviral gene expression whatsoever (29). A MARV mutant with an impaired VP35 IFN-inhibiting domain only induced IFN-α subtypes. In contrast, in the same study, Sendai virus induced multiple IFNs, including low levels of IFN-ωs. MARV infection of R06E-J cells (an Egyptian rousette embryonic cell line) led to modest induction of a few ISGs and no detectable IFN induction (62). However, a MARV isolate from a patient in Uganda did lead to significant ISG expression in RoNi/7.1 cells, though IFN-ω genes were not examined (59). These differences may be attributable to different viral strains, cell lines, and time points. Of note, all these studies were performed with immortalized cells. Even if MARV infection of these cell lines does not lead to IFN-ω induction, it is still possible that various primary cell types could serve as a source of IFN-ω *in vivo*. It has recently been shown that Egyptian rousette dendritic cells that are infected with a bat isolate of MARV upregulate IFNs and ISGs, though the few IFNω genes included in the Nanostring-based study were not reported to be significantly upregulated (63).

In conclusion, we propose that the expansion of the IFN-ω subfamily may contribute to a more flexible antiviral response that could be useful to the host by avoiding excess pathology. Ideally, this hypothesis would be tested with multiple viruses, including other viruses that naturally infect Egyptian rousettes. We provide evidence that recombinant IFN-ωs are effective against MARV infection *in vitro*. However, it remains to be determined whether our findings pertain to filovirus infection *in vivo*.

## Materials and Methods

### Cell Lines and Viruses

RoNi/7.1 cells [*Rousettus aegyptiacus* immortalized cells from kidney tissue; kindly provided by M. A. Müller and C. Drosten, Charité-Universitätsmedizin Berlin, Germany; (13)] were maintained in RoNi cell medium [Dulbecco's Modified Eagles Medium (DMEM) containing 1% MEM non-essential amino acids solution (100x concentrate), 100 units/ml penicillin, 100 μg/ml streptomycin, 1 mM sodium pyruvate] supplemented with 10% fetal bovine serum (FBS). Culture conditions for 293F suspension cells (human embryo kidney cells, ThermoFisher Scientific) have been described previously (19). Vero E6 cells (BEI Resources, Cat NR-596) were maintained in DMEM supplemented with 10% FBS, 100 units/ml penicillin and 100 μg/ml streptomycin. VSV-eGFP was a gift from Dr. John H. Connor (Boston University School of Medicine), and was propagated as previously described (19). Virus stocks for MARV isolates Musoke (GenBank: NC_001608; BEI Resources) and Angola (GenBank: KR867677.1; BEI Resources) were propagated in Vero E6 cells as described previously (64). Identity of virus extracted nucleic acids was confirmed by deep sequencing. Virus titers were determined in the same cells by plaque assay as described elsewhere (65). All work with infectious MARV was performed in the BSL-4 facility of the Texas Biomedical Research Foundation, San Antonio, TX.

### Phylogenetic and Sequence Analysis

IFN-ω genes annotated in Pavlovich et al. (19) were examined in BioEdit v7.0.0 (66). Genes were translated into protein sequences within BioEdit. Sequences were aligned with Mafft v7.305b (67) (-auto parameter), and the resulting alignment was trimmed with trimAL v1.3 (-automated1 parameter) (68). The trimmed alignments were used to generate maximum likelihood phylogenetic trees with RAxML v8.2.9 under a JTT + Γ substitution model with empirical base frequencies (69). Hundred bootstrap replicates were used to assess branch reliability. The best scoring maximum likelihood tree was analyzed in MEGA v7.0.26 (70).

To capture as many possible receptor binding sites, each protein was used as input into the NCBI conserved domain database search (https://www.ncbi.nlm.nih.gov/Structure/cdd/wrpsb.cgi) (71, 72), which relies on previous published work to pick out possible IFNAR1 and IFNAR2 binding sites (73–75). The residues at each site (20 sites for IFNAR1 and 27 for IFNAR2) were compared across all Egyptian rousette IFN-ω proteins (every protein compared to every other protein), and the total number of conserved sites divided by the total number of sites (50) is reported in Table 1 for each clade. Protein sequences were also used as input for signal sequence prediction via the Signal P v4.1 server (http://www.cbs.dtu.dk/services/SignalP/) (76). Proteins were then aligned to the human IFN-ω with ClustalW within BioEdit and the predicted signal peptides (amino acids 1–21) were cleaved. The proteins were then compared to human IFN-ω and important binding sites as described in (12) were labeled.

### Expression and Purification of Recombinant IFNs

Recombinant 6x-His tagged IFN-ω proteins and D1 were produced and characterized as previously described (19). Briefly, 293F suspension cells were transfected with plasmids encoding pCAGGS/6x-His-IFN-ω4 or IFN-ω9 (plasmids synthesized by Blue Heron Biotech, Bothell WA) according to the manufacturer's protocol using FreeStyle^TM^ MAX reagent (Thermo Fisher). D1 is domain 1 of *Bacillus anthracis* protective antigen (PA) and was expressed in *E. coli* BL21 (DE3) cells transformed with pET22b/6xHis-PA-D1. Recombinant IFNs were purified from clarified media using the Capturem His-tagged purification maxiprep kit (Clontech, Takara Bio) and buffer-exchanged into sterile PBS with a Vivaspin 2 protein concentration column (MWCO 10 kDa; GE Life Sciences). Proteins were characterized by Western blot for the 6x-His tag (anti-6x-His tag mouse mAb, Thermo Fisher), silver staining for purity (Pierce Silver Stain assay kit; Thermo Fisher), and quantified by Bradford assay (Biorad, Hercules, CA) and by 280 nm absorbance measured on a NanoDrop spectrophotometer.

### VSV Antiviral Assay

The VSV antiviral assay was performed as previously described (19). Briefly, RoNi/7.1 cells were seeded at a density of 3 × 10^4^ cells per well in 96-well plates and 1 day after seeding, were mock-treated or treated with dilutions of rIFN-ω4, rIFN-ω9, rD1, or UIFN in RoNi cell medium supplemented with 10% FBS for 4 or 8 h as indicated. Cells were infected with VSV-eGFP at an MOI of 0.1 or 0.05 (in RoNi cell medium supplemented with 2% FBS), and examined for GFP expression one day post-infection.

### MARV Antiviral Assay

RoNi/7.1 cells were seeded at a density of 3 × 10^4^ cells per well in 96-well plates ~18 h prior to treatment. Duplicate wells were mock-treated or treated with 1,000U of UIFN, 100 ng/mL of rD1, or various dilutions of rIFN-ω4 or rIFN-ω9 in RoNi cell medium supplemented with 10% FBS for 18 h. Cell supernatants were removed, and cells were mock-infected or infected with MARV Musoke or MARV Angola at an MOI of 3. After an attachment period of 1 h at 37°C, inoculum was removed and replaced with RoNi cell medium supplemented with 2% FBS, and cells were incubated for 24 h at 37°C. Cells were then inactivated and fixed with 10% formalin for 16 h, washed and stored in PBS at 4°C until use. For immunofluorescence analysis, fixed cells were permeabilized with 0.1% Triton X-100 for 5 min at room temperature, treated with 0.1 M glycine for 5 min, and incubated in blocking buffer (2% bovine serum albumin (BSA), 0.2% Tween 20, 3% glycerol and 0.05% sodium azide in PBS) for 20 min. Cells were then incubated with an anti-MARV nucleocapsid rabbit antiserum (1:500 dilution in blocking buffer) overnight at 4°C. As a secondary antibody, a goat anti-rabbit antibody conjugated to Alexa Fluor 488 (Invitrogen) was used. Cells were imaged with fluorescent microscopy and the fluorescent signal of pictures (two per sample per experiment) from two independent experiments was quantified in ImageJ.

### IFN Treatment for RNA-Seq Study

RoNi/7.1 cells were seeded at a density of 2.5 × 10^5^ cells per well in 12-well plates, and 1 day later were mock-treated or treated with 1,000U of UIFN or dilutions of rIFN-ω4, rIFN-ω9, or rD1 (0.01, 1, or 100 ng/mL) for 4 or 8 h at 37°C. Cell culture medium was removed and 600 μL of RNAzol RT (Molecular Research Center Inc, Cincinnati, OH) was added to each well. RNA in RNAzol was then transferred to an RNAse-free tube, vortexed for 20 s, and immediately stored at −80°C. Experiments were performed in triplicate, resulting in a total of 66 samples.

### RNA Isolation, Library Preparation, and Sequencing

RNA was extracted using a previously established protocol for RNAzol. Briefly, 240 μL of nuclease-free water (0.4x RNAzol volume, Ambion #AM9937) was added to each sample, followed by vigorous vortexing and pelleting of DNA and protein components. RNA was precipitated with isopropanol (equal volume) and 20 μg of glycogen (stock of 20 μg/μL, Invitrogen #10814-010), and washed twice with 75% ethanol, before resuspension in nuclease-free water. RNA was quantified by Nanodrop and a subset of samples was assessed for quality by BioAnalyzer (Agilent) evaluation on an RNA chip. All samples tested had RIN scores of 10.

1.25 μg of RNA was used as input for the TruSeq® Stranded mRNA Library Prep kit (Illumina). Briefly, mRNA was purified by polyA capture and fragmented to an average length of ~410 bases. The first strand of cDNA was synthesized with SuperScript II Reverse Transcriptase (Thermo Fisher #18064014), followed by second strand synthesis and cleanup with AMPure XP beads (Beckman Coulter #A63880). The resulting double-stranded cDNA was stored at −80°C until 3′ adenylation and end repair the following day. Samples were barcoded with adapters from the TruSeq RNA CD Index Plate (Illumina), cleaned with AMPure XP beads, and libraries were enriched by PCR for 12 cycles. Final libraries were washed twice with AMPureXP beads (final bead wash ratio: 0.85x to remove adapter dimers), and quantified by Qubit 3.0 assay. A subset of samples was examined by DNA Bioanalyzer (Agilent) for quality purposes before pooling samples within each of three replicates. Pooled libraries were sent to Tufts Genomics Center for size selection (pippin size selection, 180–1,100 bp) and sequencing. Each library was sequenced on a separate lane of an eight-lane flow cell in high output mode on an Illumina HiSeq 2500 using single-end 100 bp chemistry.

### RNA-Seq Differential Expression Analysis

Raw reads were demultiplexed by the Tufts Genomics Center and evaluated for quality with FastQC v0.11.3. Remaining 5′ adapter sequences were trimmed using cutadapt v1.5, and all reads shorter than 50 bp were discarded. Trimmed reads were mapped to the Raegyp2.0 genome (RefSeq accession: GCF_001466805.2) with hisat2 v2.1.0 (77, 78), with an average mapping rate of 97.1%. Count tables of uniquely mapped reads were tabulated with HTSeq v0.6.1p1 (79) with the parameters –stranded=reverse and –mode=union for htseq-count with a gtf annotation file from RefSeq (with in-house modifications to use GeneID as the ID attribute). Count tables were used for pairwise differential expression analysis (and multiple hypothesis testing correction) with edgeR (80, 81) within the R environment (R version 3.4.3). First, an ANOVA-like test was performed where each treatment (rD1, rIFN-ω4, rIFN-ω9) was compared to all other treatments for a given treatment concentration and time.

For all genes rejecting the null in the ANOVA-like test with an FDR ≤ 0.05 (Benjamini-Hochberg procedure), each IFN-treated sample was compared to the corresponding rD1-treated sample at the same concentration and time point (e.g., treatment with 1 ng/mL of rIFN-ω4 for 4 h compared to treatment with 1 ng/mL of rD1 for 4 h), and IFN-ω4 was compared to IFN-ω9 for the same conditions. Genes were considered differentially expressed if the *p*-value of the pairwise comparison was <0.05/3 and if the absolute value of the log_2_ fold change in the pairwise comparison was >1. UIFN-treated samples were compared to untreated samples at the same time point, with an FDR < 0.05 and the absolute value of the log_2_ fold change was >1. Gene symbols were mapped back onto Gene IDs with the rentrez package in R. Plots were generated in R with the pheatmap (82) and ggplot2 packages, except for Venn diagrams, which were produced in Venn diagram plotter (v1.5.5228.29250).

### ISG Database Analysis

Genes that were upregulated at any time point and concentration for each treatment (post ANOVA, post pairwise analysis) were searched against the Interferome (v2.01) database (27), data from Shaw et al. (25) (accessible at http://isg.data.cvr.ac.uk/), and data from Mostafavi et al. (26). Genes without canonical gene symbols were cross-referenced with the NCBI Gene database to identify probable gene names based on the given gene description. Alternate gene names were identified using UniProt and Genecards. Genes were considered uncharacterized if they were annotated as uncharacterized by the NCBI annotation pipeline based on insufficient homology to any other gene in Genbank.

## Data Availability Statement

The data discussed in this publication have been deposited in NCBI's Gene Expression Omnibus (83) and are accessible through GEO Series accession number GSE145761 (https://www.ncbi.nlm.nih.gov/geo/query/acc.cgi?acc=%GSE145761).

## Author Contributions

SP, TK, and EM: experimental design (RNA-sequencing). SP, FF, and AH: technical design (RNA-sequencing, VSV). SP: experimental execution (RNA-sequencing, VSV). SP, EM, and RD: experimental design (MARV). TD: experimental execution (MARV). SP and TK: data analysis. SP, EM, and TK: manuscript writing. All authors: manuscript editing.

### Conflict of Interest

The authors declare that the research was conducted in the absence of any commercial or financial relationships that could be construed as a potential conflict of interest.

## Supplementary Material

Figure S1 Change in ISG expression over time. **(A)** Genes that were significantly differentially expressed in cells treated with 1 or 100 ng/mL rIFN-ω4 or rIFN-ω9. Only upregulated genes with *p* ≤ 0.05/3 in the pairwise test were included if expressed after both rIFN-ω4 and rIFN-ω9 treatment, regardless of treatment time. **(B)** Pattern of differential expression over time. Genes that passed the significance criteria for the ANOVA tests for a given concentration of IFN at any time point are shown.

Figure S2 Purity of rIFN-ω4, rIFN-ω9, and rD1 preparations containing 6xHis-tagged recombinant proteins. Recombinant proteins from clarified 293F cell supernatants were purified using affinity chromatography, dialyzed into PBS, quantified by Nanodrop and Bradford assay, and evaluated by **(A)** silver stain for purity (100 ng/well), and by **(B)**. Western blot for His-tag specificity (250 ng/well).

Table S1 Differentially expressed genes in post-ANOVA and pairwise tests.

Table S2 Upregulated genes in the ISG databases.
