# Supplementary figures and images for: Egyptian Rousette IFN-ω Subtypes Elicit Distinct Antiviral Effects and Transcriptional Responses in Conspecific Cells
Source: Front Immunol. 2020 Mar 13;11:435. doi: 10.3389/fimmu.2020.00435 (PMC7083018; doi:10.3389/fimmu.2020.00435)

**A**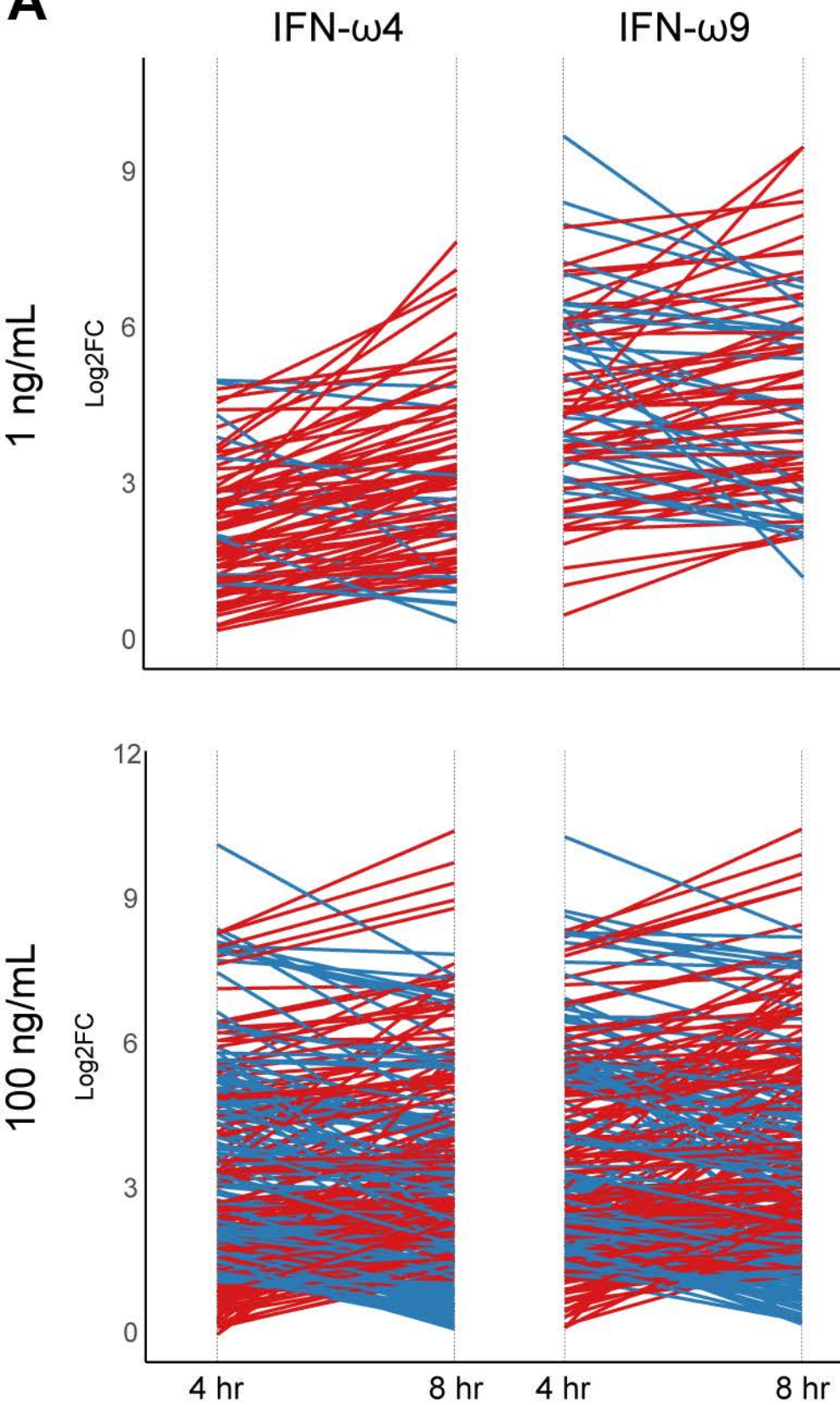**B**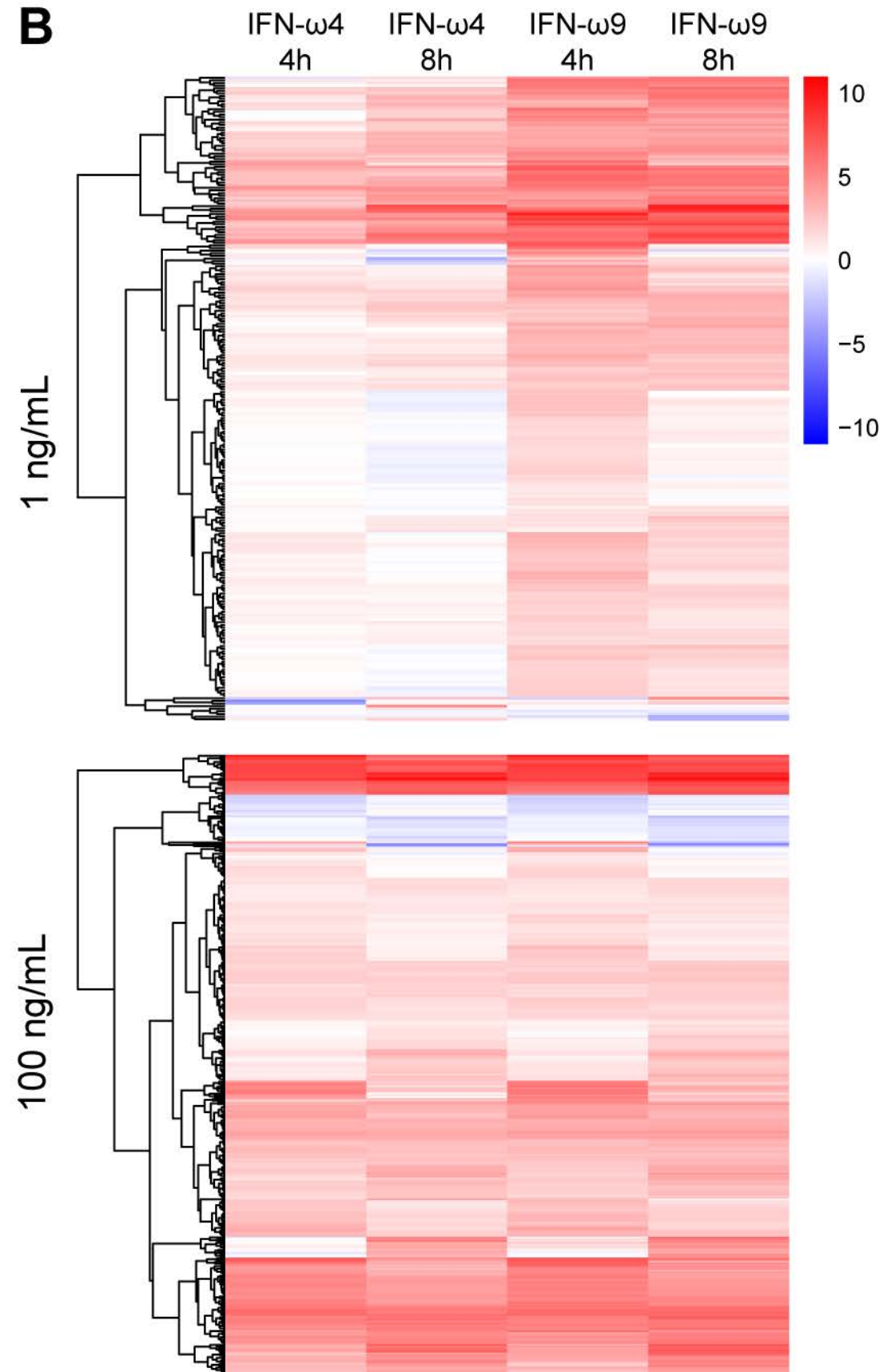

Supplement: Figure S1 — Change in ISG expression over time. (A) Genes that were significantly differentially expressed in cells treated with 1 or 100 ng/mL rIFN-ω4 or rIFN-ω9. Only upregulated genes with p ≤ 0.05/3 in the pairwise test were included if expressed after both rIFN-ω4 and rIFN-ω9 treatment, regardless of treatment time. (B) Pattern of differential expression over time. Genes that passed the significance criteria for the ANOVA tests for a given concentration of IFN at any time point are shown. [file Data_Sheet_1.PDF]

**A**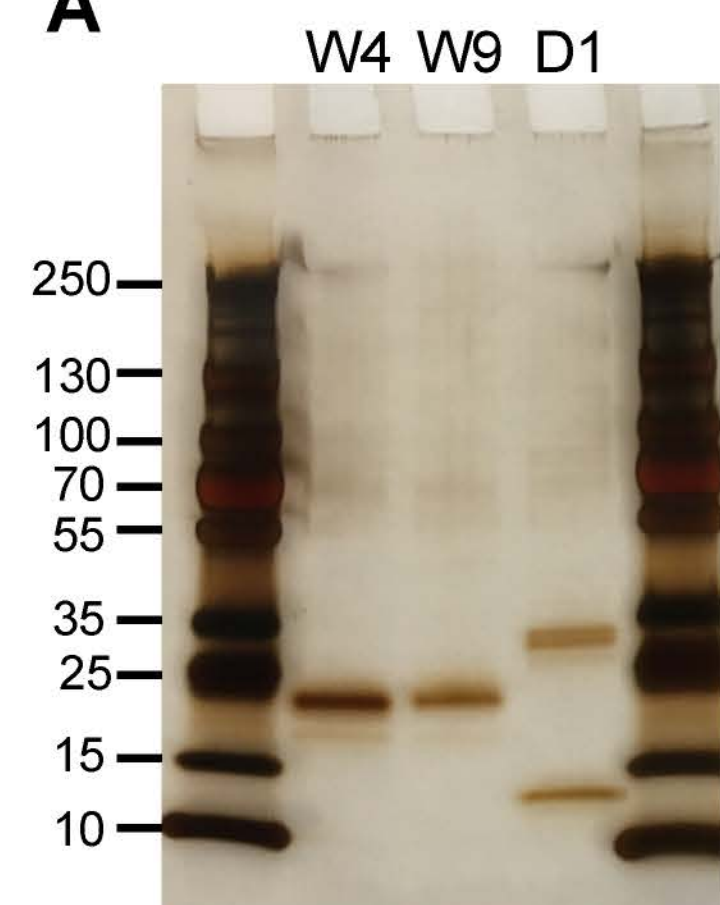**B**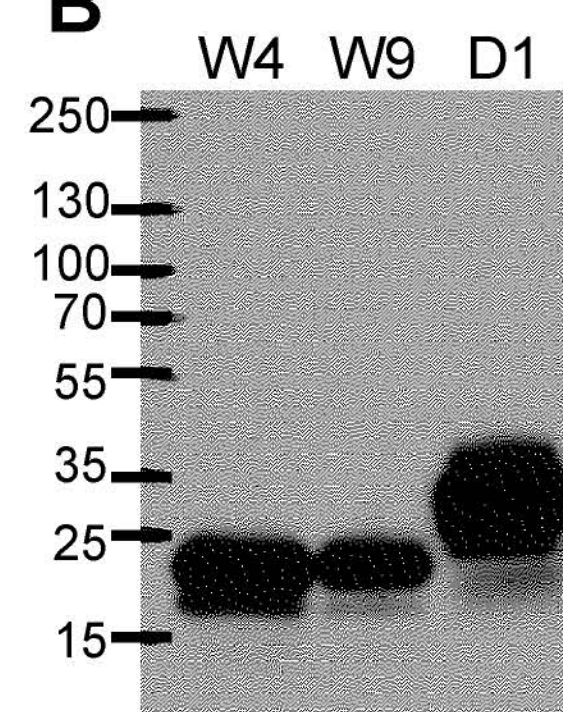

Supplement: Figure S2 — Purity of rIFN-ω4, rIFN-ω9, and rD1 preparations containing 6xHis-tagged recombinant proteins. Recombinant proteins from clarified 293F cell supernatants were purified using affinity chromatography, dialyzed into PBS, quantified by Nanodrop and Bradford assay, and evaluated by (A) silver stain for purity (100 ng/well), and by (B). Western blot for His-tag specificity (250 ng/well). [file Data_Sheet_2.PDF]
